# HPV integration hijacks and multimerizes a cellular enhancer to generate a viral-cellular super-enhancer that drives high viral oncogene expression

**DOI:** 10.1371/journal.pgen.1007179

**Published:** 2018-01-24

**Authors:** Alix Warburton, Catherine J. Redmond, Katharine E. Dooley, Haiqing Fu, Maura L. Gillison, Keiko Akagi, David E. Symer, Mirit I. Aladjem, Alison A. McBride

**Affiliations:** 1 Laboratory of Viral Diseases, National Institute of Allergy and Infectious Diseases, National Institutes of Health, Bethesda, Maryland, United States of America; 2 Developmental Therapeutics Branch, National Cancer Institute, National Institutes of Health, Bethesda, Maryland, United States of America; 3 Department of Internal Medicine, The Ohio State University, Columbus, Ohio, United States of America; 4 Department of Cancer Biology and Genetics, The Ohio State University Comprehensive Cancer Center, Columbus, Ohio, United States of America; 5 Human Cancer Genetics Program, Department of Cancer Biology and Genetics, The Ohio State University Comprehensive Cancer Center, Columbus, Ohio, United States of America; 6 Department of Biomedical Informatics (adjunct), The Ohio State University Comprehensive Cancer Center, Columbus, Ohio, United States of America; Gladstone Institute of Virology and Immunology, UNITED STATES

## Abstract

Integration of human papillomavirus (HPV) genomes into cellular chromatin is common in HPV-associated cancers. Integration is random, and each site is unique depending on how and where the virus integrates. We recently showed that tandemly integrated HPV16 could result in the formation of a super-enhancer-like element that drives transcription of the viral oncogenes. Here, we characterize the chromatin landscape and genomic architecture of this integration locus to elucidate the mechanisms that promoted *de novo* super-enhancer formation. Using next-generation sequencing and molecular combing/fiber-FISH, we show that ~26 copies of HPV16 are integrated into an intergenic region of chromosome 2p23.2, interspersed with 25 kb of amplified, flanking cellular DNA. This interspersed, co-amplified viral-host pattern is frequent in HPV-associated cancers and here we designate it as Type III integration. An abundant viral-cellular fusion transcript encoding the viral *E6/E7* oncogenes is expressed from the integration locus and the chromatin encompassing both the viral enhancer and a region in the adjacent amplified cellular sequences is strongly enriched in the super-enhancer markers H3K27ac and Brd4. Notably, the peak in the amplified cellular sequence corresponds to an epithelial-cell-type specific enhancer. Thus, HPV16 integration generated a super-enhancer-like element composed of tandem interspersed copies of the viral upstream regulatory region and a cellular enhancer, to drive high levels of oncogene expression.

## Author summary

Oncogenic human papillomavirus (HPV) infection is responsible for ~5% human cancers. A key event in the development of many of these cancers is integration of the viral genome into host chromatin. Integration results in dysregulated expression of the viral oncogenes, which promotes unregulated cellular division and the accumulation of cellular mutations, ultimately leading to cancer. Genetic rearrangement and/or amplification of cellular sequences flanking sites of integration are frequent in HPV positive tumors, and this can influence transcription of the viral oncogenes from integrated loci. We show an example where the HPV16 genome integrated adjacent to a cellular, tissue-specific enhancer and subsequently the viral DNA and flanking cellular sequences were co-amplified into a tandemly interspersed viral-cellular sequence array. The genetic and epigenetic signature of this site promoted the formation of a *de novo* super-enhancer-like element to drive high viral oncogene expression. This provides insight into the genesis of super-enhancer-like elements and is a novel mechanism by which HPV integration can promote oncogenesis.

## Introduction

High oncogenic-risk human papillomaviruses (HPVs) are the etiological agent responsible for virtually all cervical cancers [1], and a growing number of other anogenital and oropharyngeal cancers [2]. During the normal viral life cycle, HPVs are maintained as extrachromosomal elements within the cell nucleus. However, during persistent infection with oncogenic HPVs the viral genome can become accidently integrated into the host chromatin [3]; this is a key event in many HPV-associated cancers. For the most part, HPV integration sites are randomly distributed throughout the host genome but preferentially associate with common fragile sites [4, 5] and transcriptionally active regions [6, 7]. The viral genome is usually found integrated as either a single copy or multiple, tandem, head-to-tail repeats at a single locus within the host genome [8]. In addition, there are often associated focal amplifications and rearrangements of the surrounding cellular flanking sequences [9–12]. These features result in a range of integration sites that are unique in terms of their genetic/epigenetic composition and transcriptional potential.

There are several mechanisms through which HPV integration can promote oncogenesis (reviewed in [13]). Often there is disruption of the viral *E2* gene, which eliminates its repressive function over the *E6/E7* oncogenes [3]. Dysregulation of E6/E7 expression promotes cellular proliferation, abolishes cell cycle checkpoints and induces progressive genetic instability at the integrated locus [14]. Insertional breakpoints can also occur within the *E1* gene, eliminating downstream E2 function and the growth suppressive properties of the E1 protein [15, 16]. Furthermore, upon integration, the viral *E6/E7* oncogenes are expressed from the early viral promoter as a viral-host fusion transcript [17, 18]. Such fusion transcripts have increased stability, resulting in increased *E6/E7* oncogene expression compared to that expressed from extrachromosomal HPV genomes [8]. Less frequently, HPV integration occurs within host cancer-associated gene loci or pathways and modifies target gene expression [10, 11, 19–21], which can further support neoplastic progression.

We have recently demonstrated that tandemly integrated HPV16 in the cervical-derived cell line 20861 can form a Brd4-dependent super-enhancer-like element [22]; this is a novel mechanism that drives viral oncogene expression from the integrated locus. Super-enhancers are large clusters of enhancer elements that are highly occupied by the transcriptional machinery and often develop to drive oncogene expression in cancer [23–26]. Treatment of 20861 cells with BET-inhibitors disrupts Brd4 binding to this *de novo* super-enhancer, resulting in downregulation of *E6/E7* and induction of cellular senescence [22].

In this follow up study, we have characterized the genomic architecture of the integration site in 20861 cells to elucidate the genetic and epigenetic mechanisms operating at this locus. Using whole genome sequencing (WGS), hybrid capture sequencing, RNA-seq, ChIP-seq and fiber-FISH (fluorescent in situ hybridization), we show that the HPV16 genome is integrated into an intergenic region in chromosome 2 and both the viral genome and approximately 25 kb of flanking cellular DNA are amplified to about 26 copies. The combination of the viral upstream regulatory region (URR) and a basal cellular enhancer in the flanking cellular sequence results in strong enrichment of super-enhancer markers at the integration locus and high expression of a fusion transcript encoding E6/E7, which is spliced into a cryptic acceptor site in the cellular sequence. This hijacking of a basal cellular enhancer, resulting in the formation of a viral-cellular super-enhancer, is a novel mechanism by which HPV integration can promote neoplasia.

## Results

### Characterization of the viral integration site in 20861 cells

The original W12 cell line, derived from a cervical intraepithelial lesion, contained mostly extrachromosomally replicating HPV16 genomes [8]. However, cells containing integrated HPV16 genomes were found to frequently outgrow the culture and so the Lambert laboratory isolated a series of clones that contained either extrachromosomal or integrated viral genomes [8]. One of these clones, 20861, has been well characterized and is frequently used as a representative cell line with integrated HPV16 DNA, while the 20863 clone contains extrachromosomal genomes [8, 17]. We have recently shown that viral oncogene expression in 20861 cells is driven by a super-enhancer-like element [22].

**Fig 1:**
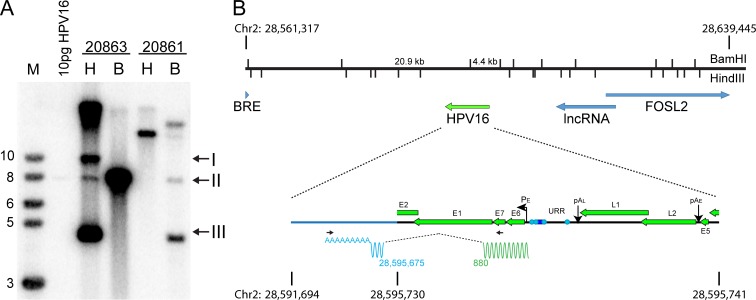
20861 cells have multiple, rearranged copies of HPV16 at the integration site. **A,** Southern blot analysis of 20861 and 20863 W12 cell lines. Genomic DNA was digested with either HindIII (H; does not cut HPV16 DNA) or BamH1 (B; a single cut in HPV16 DNA). DNA fragments were separated by electrophoresis, transferred to membranes and probed with 32P-labeled HPV16 DNA. Arrows indicate relaxed circle (I), unit length linear (II), and supercoiled HPV16 genomes (III). Viral copy number was quantified by comparison to 10 pg linearized HPV16 DNA. The position of BamHI and HindIII cleavage sites spanning the integrated locus (chr2:28,561,317–28,639,445; hg19) and expected band sizes from Southern blot are shown in **B**. **B,** Diagram of the integration locus of HPV16 in 20861 cells. APOT analysis showed that HPV16 was integrated into chr2 p23.2 and expresses an E6/E7 fusion transcript that splices to an exon located at 28,595,425–28,595,675 (hg19). The wavy line underneath the expanded viral genome represents the fusion transcript. Primer positions are denoted by black horizontal arrows. Green represents viral sequences, and blue represents host-derived sequences. Splice donor (viral) and acceptor (host) sites are indicated below.

Here we further characterize the HPV16 integration site in 20861 cells in several ways. First, the overall arrangement of the locus was analyzed by Southern blot. Total genomic DNA was extracted from 20861 and 20863 cells. The DNA was digested with either HindIII, which does not cut the HPV16 genome and therefore allows visualization of the supercoiled and relaxed circle forms of the extrachromosomal genome, or with BamHI, which linearizes the viral DNA. As shown in Fig 1A, 20863 showed the expected forms of extrachromosomal DNA. However, the pattern of bands observed in 20861 DNA indicated that there was rearrangement of viral and cellular DNA at the integration locus, as almost no unit length genomes were observed. The observed pattern is virtually identical to the original analysis of the W12 clones determined by Lambert and colleagues [8]. Quantitation of the Southern blot shown in Fig 1A determined that there were ~24 copies of the integrated viral genome in 20861 cells and ~230 extrachromosomal HPV16 copies in 20863 cells, similar to the copy numbers previously defined [8, 22].

To determine the location of the integration site within the cellular genome, we initially used a technique called Amplification of Papillomavirus Oncogene Transcripts (APOT). This technique amplifies sequences derived from polyadenylated RNA consisting of viral E6/E7 sequences spliced to host derived transcripts [27]. RNA was isolated from 20861 cells and reverse transcribed using an oligo-dT primer. The viral-host fusion transcript was amplified from the cDNA by PCR using forward primers from the E6/E7 region and oligo-dT reverse primers, and the resulting PCR products were purified and sequenced. As shown in Fig 1B, this identified a fusion transcript that had spliced into a cryptic acceptor at chr2: 28,595,675 (hg19). The cellular exon spanned nucleotides 28,595,425–28,595,675 and did not align with any annotated genes, or contain an open reading frame (ORF). The size of the fusion transcript was shorter than that observed by Jeon and colleagues using Northern blot analysis [8] and, as we show below, is a minor, shorter RNA species that uses the same viral-host splice sites.

### The cellular integration locus is co-amplified with HPV16

**Fig 2:**
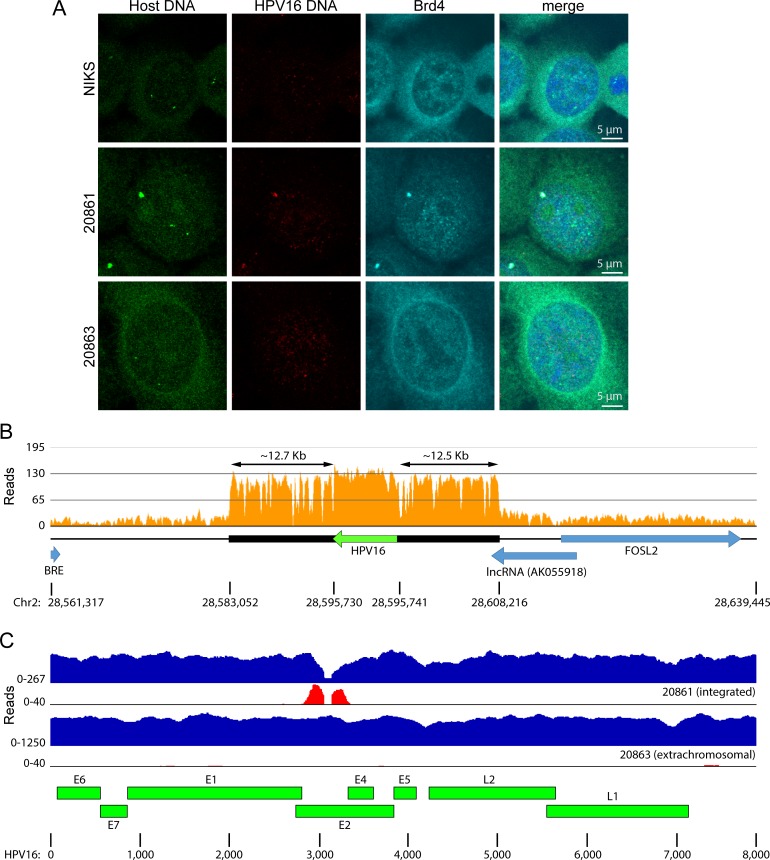
Cellular DNA at the integration region is co-amplified with HPV. **A,** The location of Brd4, HPV16 DNA, and cellular DNA flanking the chromosome 2 integration site were studied in W12 20861 and 20863 (extrachromosomal HPV16) cells, and in HPV-negative NIKS human keratinocyte cells by combined immunofluorescence /fluorescent in situ hybridization. HPV16 DNA is shown in red, and the flanking cellular sequence is shown in green. Brd4 signal is in cyan and nuclei are counterstained with DAPI (blue). In comparable FISH experiments, about 70% 20861 cells contained large focus of signal from the cellular flanking sequence that colocalized with the HPV16 signal. **B,** Alignment of 20861 WGS data to the human reference genome (hg19) showed focal amplification of cellular sequence at the HPV16 integration site. Histograms represent depth of coverage of aligned reads (quality score threshold, 30). Amplified region is marked by a black horizontal bar; HPV16 genome and cellular genes are represented by green and blue horizontal arrows, respectively. **C,** WGS reads were aligned to the HPV type 16 isolate 16W12E (AF125673.1) reference genome. Histograms represent copy numbers of viral sequences (blue) and counts of discordant paired-end reads supporting insertional breakpoints (red). The scale (y-axis) of each plot was normalized to maximum read counts. HPV breakpoints are defined further in Table 1 and S1 Table.

To verify the location of the viral integration site, a FISH probe was generated from a cellular BAC clone RP11-1140H22 (chr2: 28,504,596–28,660,966; hg19), which spanned the integration locus. As shown in Fig 2A, hybridization of 20861 cells with the cellular genomic DNA BAC probe, along with an HPV16 probe, revealed that both probes colocalized in >70% of cells. As expected, the cellular probe gave rise to two signals in HPV-negative NIKS human keratinocyte control cells. However, the signal that overlapped the HPV16 integration locus in 20861 cells was far stronger. This indicated that cellular sequences flanking the HPV16 integration site had been co-amplified along with the viral genome. In addition, Brd4 colocalized with the 20861 HPV integration site as previously shown [22]. To confirm the amplification of cellular sequences flanking the integration site, we used quantitative PCR (qPCR) to measure the copy number of the sequences flanking the HPV16 integration site relative to the *ACTB* gene. This showed that there were approximately 20 copies of the region flanking the integration site relative to HPV16 in W12 20861 cells (Supplementary S6D Fig). Therefore, cellular sequences flanking the integration sites are co-amplified along with the HPV16 sequences.

To define the amplification at the HPV16 integration site in 20861 cells, 2 x 150 bp paired-end libraries were generated from total genomic DNA and subjected to WGS using the HiSeq 4000 platform (Illumina Genome Network). WGS data were aligned to the human reference genome (hg19) with ~7X sequencing coverage (Table 1). This showed a 12 bp deletion at chr2: 28,595,730–28,595,741 that marked the main HPV insertional breakpoints (Supplementary S1 Table). The flanking amplified cellular sequence spanned 25.2 kb (chr2: 28,583,052–28,608,216; approximately 12.5 kb either side of the integrated viral genome), and overlapped with 941 bp of exon 2 of the human non-coding RNA AK055918 (Fig 2B). The depth of coverage over this region was increased 28-fold along with the integrated HPV16 genome (very similar to the 24–26 copies of viral genome measured by Southern blot analysis and qPCR). We identified a gap in the aligned HPV sequence reads in the WGS analysis of the cell line 20861, within the *E2* gene (Fig 2C). This gap was not seen in the 20863 cells derived from the same parental W12 cells. The boundaries of this coverage gap defined a 101 bp deletion at the HPV genome nt position 3052–3152 (Fig 2C). We also observed a minor fraction of sequence reads in 20861 cells that still aligned to these deleted HPV sequences, suggesting that those viral sequences originated from a different, minor integration site (see below, Supplementary S1 Table, and S1 Fig).

**Table 1:** Summary of WGS sequencing of W12 clonal cell lines.

W12 Sub-clone	Hg19 sequencing depth of coverage	HPV16 sequencing depth of coverage	HPV16 copy number per cell	# HPV16 insertional breakpoints	HPV16 copy number per cell determined by qPCR
20861	7.73	201.94	26.12	2	25.96
20863	7.77	1,022.37	131.57	0	142.25
20831	7.53	59.51	7.90	1	7.90
20862	7.15	119.03	16.66	0	18.41

### Hybrid capture sequencing of 20861 integration site

To confirm the precise viral-host junctions, we re-sequenced the viral and viral-host insertional breakpoint sequences in 20861 and 20863 genomic DNA, using custom hybrid capture baits (Supplementary S1 Table). This much more sensitive approach verified that the major HPV16 insertional breakpoints were at chr2:28,595,730 and at chr2:28,595,741. The viral-host fusion reads confirmed that the viral genome was integrated within the *E2* gene at the position of the deletion (HPV genome nt position 3052–3152). The reads also confirmed the 12 bp deletion at the host insertion site in chr2 (28,595,730–28,595,741).

Additional viral insertional breakpoints were detected in both 20861 and 20863 reads, but these sequences were only supported by a few reads each (out of many millions of reads per sample), suggesting that they were minor integration sites likely representing minor sub-clonal cell populations (Supplementary S1 Table). Another explanation for their relatively low counts was that they represented non-specific technical artifacts related to the very sensitive hybrid capture method, PCR amplification of libraries, or deep sequencing of the libraries.

### HPV16 genome copies are interspersed with cellular DNA

**Fig 3:**
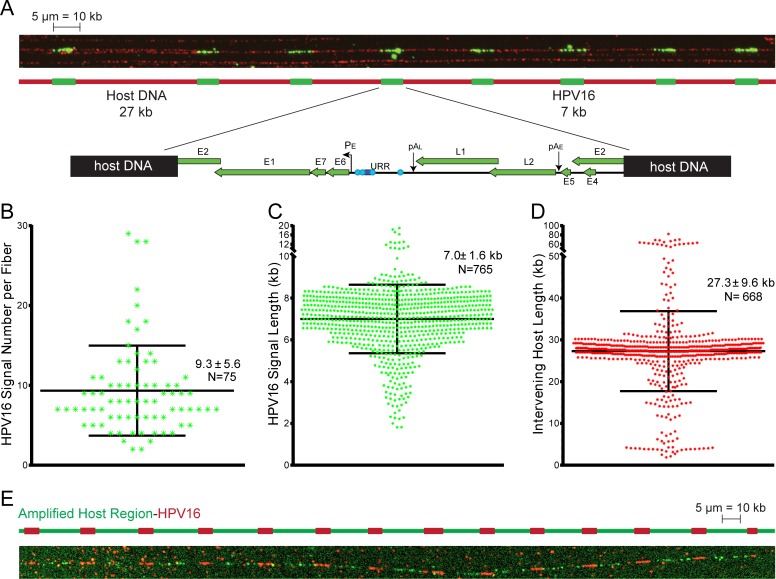
Analysis of the 20861 integration site by molecular combing. **A,** Representative image of a DNA fiber containing the Type III HPV16 integration site in 20861 cells. The HPV16 DNA signal is shown in green, and the DNA backbone was visualized with a single-stranded DNA (ssDNA) antibody, shown in red. The map shows the breakpoint of HPV16 in the E2 ORF in the 20861 Type III integration site. **B,** Scatter plot showing the number of HPV16 genomes per individual DNA fiber. Average signal count per fiber and SD is shown, along with total number of fiber counts. Data are from two replicates. **C-D,** Scatter plots showing measured HPV16 signal length (C), and measured length of the interspersing space between HPV16 signals from every fiber (D). Measurements were based on the conversion 1 μm = 2 kb. Average measured length in kb and SD is shown, along with total number of fibers counted. Data are from two replicates. **E,** Two-color fiber-FISH in 20861 cells using DNA probes against HPV16 (shown in red) and the amplified cellular sequence identified from whole genome sequencing (shown in green) confirm the organization of the 20861 Type III integration site as predicted from sequence analysis. Data are from two biological replicates.

Integration events can be classified as Type I, in which a single viral genome is found integrated into the host DNA; or Type II, in which multiple tandemly repeated viral genomes are integrated at a single locus [8]. It has been proposed previously that approximately 30 copies of HPV16 are tandemly integrated at a single locus in 20861 cells in what was presumed to be a Type II pattern [8, 17]. However, we show in Fig 2 that there is co-amplification of the flanking cellular sequences to a copy number similar to that of the integrated HPV16 genome. To further determine the organization of the integration site, we performed molecular combing/fiber-FISH to detect HPV16 genomes on stretched, individual DNA molecules. As shown in Fig 3, measurement of linear fluorescent signals showed that the HPV16 genome was repeated in tandem units with interspersing cellular DNA of about 27 kb, in good agreement with the 25.2 kb amplified region measured by sequencing. We have designated this integration pattern of interspersed viral and host DNA as a Type III integration event, to distinguish it from Type II events in which no intervening host sequence is present. Analysis of 75 independent DNA fibers containing the Type III 20861 integration site showed that up to 28 copies of interspersed viral DNA could be detected on a single fiber (Fig 3B). Given the low probability of combing a complete integration site, the largest repeated number of genomes (28 copies, N = 2) is likely to be the actual size of the site, and is consistent with our genomic sequencing data. The signal length of the integrated HPV16 genome measured on average 7.0 kb ± 1.6 kb (Fig 3C), whilst the intervening space between the tandemly repeated viral copies measured approximately 27.3 kb ± 9.6 kb (Fig 3D). Discrepancies in unit length measurements across individual fibers can be accounted for by the inherent limit of resolution for this technique, which is about 1 kb [28].

The unit length of the intervening host sequence closely matched the 25.2 kb amplified cellular region identified from WGS, suggesting that the region flanking the integration site had amplified into a viral-host concatemer. To confirm this, and further validate the location of the integration site within chromosome 2, two-color fiber-FISH was performed on the combed DNA strands [29]. Using DNA probes against HPV16 (red signal), and the amplified region at chr2: 28,583,052–28,608,216 (green signal), we confirmed that the repeating unit was indeed a viral-host concatemer (Fig 3E).

In addition to the major Type III integration event represented in Fig 3, we observed a second integration pattern in 20861 cells that appeared to be a Type II integration event, as it contained tandemly integrated HPV16 genomes without interspersed cellular sequence (Supplementary S1 Fig). This integration pattern accounted for ~30% of the total number of DNA fibers containing integrated HPV16 compared to the Type III integration event. The location of this integration event is not clear, but it is likely close to the site on chromosome 2 (see Supplementary S1 Table) as there are no other prominent foci apparent by FISH of interphase cells (Fig 2A).

### Transcription from the 20861 integration site

**Fig 4:**
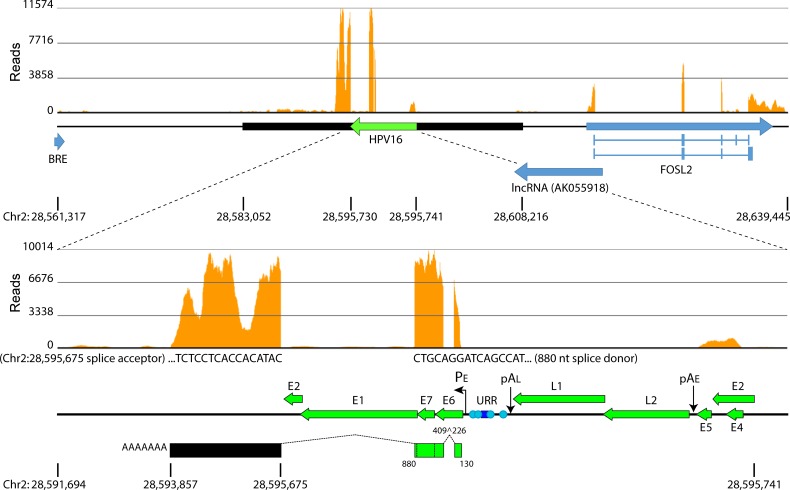
Transcription from the integrated locus in 20861 cells. Alignment of RNA-seq reads to the human (hg19) and HPV type 16 isolate 16W12E (AF125673.1) reference genomes showed high expression of a fusion transcript encoding E6/E7 spliced into a cryptic acceptor (chr2: 28,595,675). Histograms represent depth of coverage of aligned reads. Amplified region is marked by a black horizontal bar; HPV16 genome and cellular genes are represented by green and blue horizontal arrows, respectively. Schematic of the viral-cellular fusion transcript is depicted in the lower panel; encoded viral nucleotides and splice sites are indicated. Data are averaged from three biological replicates.

To further address transcription from the integrated locus we performed RNA-seq analysis and aligned sequencing reads to a custom reference assembly that contained the inverted HPV16 reference sequence incorporated at the junctional breakpoints identified from WGS. Alignment of RNA-seq reads to both the human (hg19) and HPV type 16 isolate 16W12E (AF125673.1) reference genomes [30] showed high expression of a fusion transcript spliced into the same acceptor site (chr2: 28,595,675; hg19) as that identified from APOT (Fig 4). Alignment of reads to the host sequence showed three strong peaks. The largest mRNA species was approximately 2.4 kb, >1.5 kb longer than the transcript isolated from APOT, which corresponded to the first cellular peak, but similar in size to the transcript first noted by Jeon and colleagues using Northern blot analysis [8]. This suggests that several RNA species which utilize the same viral-host splice sites are encoded by the Type III integration site in 20861 cells.

### Chromatin profile of the 20861 integration site

**Fig 5:**
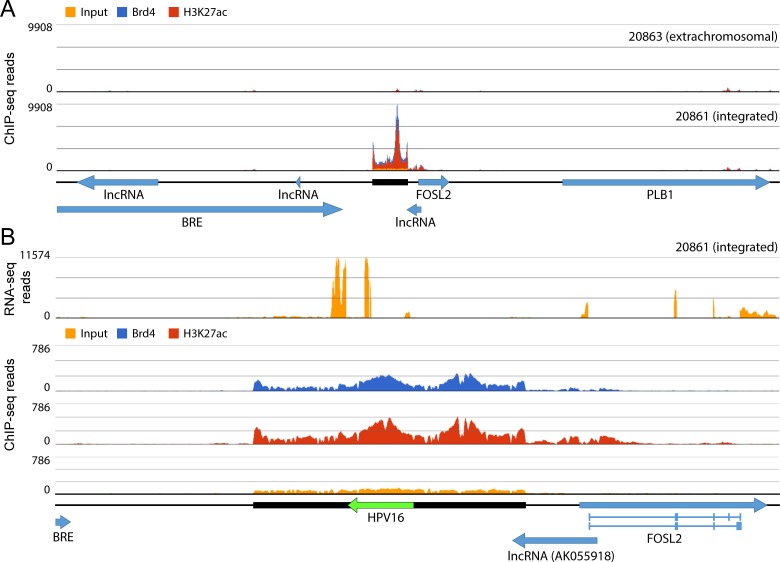
Super-enhancer markers are enriched over the integration site in 20861 cells. **A,** Alignment of ChIP-seq reads to the human reference genome (hg19) in 20861 and 20863 cells. Data are averaged from two biological replicates. **B,** Alignment of ChIP-seq reads to human and HPV type 16 isolate 16W12E (AF125673.1) reference genomes showed enrichment of super-enhancer markers over both viral and cellular chromatin. Histograms represent depth of coverage of aligned reads. Amplified region is marked by a black horizontal bar; HPV16 genome and cellular genes are represented by green and blue horizontal arrows, respectively. Data are averaged from two biological replicates.

We have shown previously that integration of HPV16 in 20861 cells results in the formation of a super-enhancer-like element that drives viral E6/E7 mRNA expression from the region [22]. This element was originally detected because of extremely high levels of H3K27ac and binding of MED1 and Brd4 at the HPV integration locus. To address exactly where these super-enhancer markers were binding with respect to the integrated viral genome and flanking cellular sequences, we performed ChIP-seq analysis using antibodies against Brd4 and H3K27ac. Alignment of paired-end reads immunoprecipitated with these antibodies to the human reference genome (hg19) showed strong enrichment of Brd4 and H3K27ac signals adjacent to the integration locus in 20861 cells relative to levels observed in 20863 cells, which do not contain integrated HPV16 at this locus (Fig 5A). To address enrichment patterns across the entire integrated locus (viral and cellular sequences) in 20861 cells, we aligned the ChIP-seq reads to our custom reference assembly of the integration locus. This showed very strong enrichment of the super-enhancer markers across both viral and cellular sequences (Fig 5B), even after accounting for amplification of this region. Two peaks were observed, one that was centered on the viral URR and another in the cellular flanking sequence about 6 kb upstream. Analysis of the same cellular region in 20863 cells showed only minimal enrichment of H3K27ac at this locus.

### Enrichment of super-enhancer markers over the viral URR is not observed in other integrated W12 sub-clones

To determine whether there was similar enrichment of the super-enhancer markers at other HPV16 integration sites we examined in detail two additional W12 clones, 20831 and 20862. We generated and analyzed WGS data at comparable depths of sequencing coverage for these additional clones (see Table 1 and Supplementary S2 Fig). These clones were originally reported to contain approximately 60 copies of tandemly integrated HPV16 [8, 17], however WGS and qPCR (Table 1) indicated a lower copy number, suggesting that viral genome copies had been lost over time.

**Fig 6:**
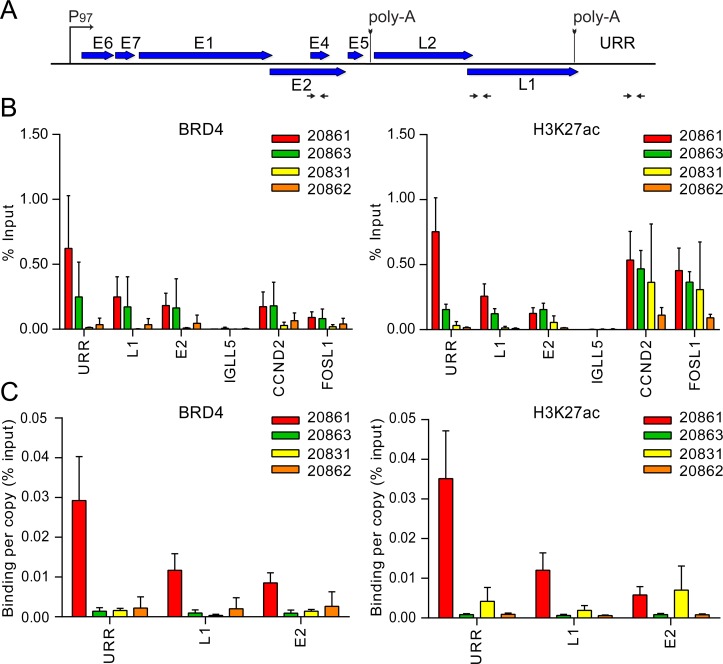
Super-enhancer markers are enriched over the viral URR in 20861, but not other integrated W12 sub-clones. Chromatin immunoprecipitation (ChIP) was performed in 20861, 20863, 20831 and 20862 cells using antibodies against Brd4 and H3K27ac. **A,** Map of linearized HPV16 genome showing primer positions (denoted by black horizontal arrows) for the upstream regulatory region (URR), L1 and E2. **B,** ChIP DNA samples were analyzed by real-time qPCR using primers against target promoters, indicated in panel A. ChIP signals were expressed as the percentage of immunoprecipitated chromatin DNA relative to the total amount of input chromatin (% Input). CCND2 and FOSL1 were included as positive controls for super-enhancer loci; IGLL5 was included as a negative control for Brd4 binding in these cells. **C,** To account for variations in viral copy number between W12 cells, ChIP signals were expressed as binding per single-copy genome. Background signal at each locus (measured by no-antibody controls) was subtracted from corresponding ChIP signals. Average binding levels were calculated from three independent experiments. Error bars represent SD. Note that similar experiments were previously conducted on 20861 and 20863 cells (using different datasets) [22].

Chromatin immunoprecipitation (ChIP) followed by qPCR showed that strong enrichment of super-enhancer markers over the viral URR was not observed in 20831 and 20862 cells (Fig 6), and, unlike in 20861 cells, the prominent nuclear BRD4 focus was not characteristic of these cells by immunofluorescence (Supplementary S3 Fig). We propose that super-enhancer function is likely dependent on the genetic and epigenetic architecture of the locus. The 20831 and 20862 cells were also subjected to RNA-seq and ChIP-seq with Brd4 and H3K27ac antibodies. This revealed that in both cell lines the virus was integrated at HPV16:3740-chr3:189,662,800, indicating that this integration event likely occurred prior to the isolation of individual clones. This is consistent with the studies of Jeon and Lambert that indicated that these clones had very similar properties [8, 17]. This Type III integration site was not present in the 20861 or 20863 cells, however (Supplementary S1 Table). Additional integration breakpoints were undetectable in 20831 and 20862 cells through WGS. Unlike 20861 and 20863 cells, these clones were not subjected to hybrid capture.

Fiber-FISH analysis of 20831 and 20862 cells showed that, as with 20861 cells, both cell lines contained Type II and Type III integration patterns (Supplementary S4 and S5 Figs). This was further substantiated by Southern blot analysis (shown in Supplementary S6 Fig). When digested with a restriction enzyme that cleaves the viral genome once, Type II integration gives a viral genome unit length of ~7.9 kb, while Type III integration results in bands comprised of the viral-host junctional sequences. Quantitation of the Type II and III fragments gives an approximation of the relative proportion of viral copies at each integration site. A combination of Southern blot analysis, WGS and qPCR of viral sequences demonstrated that the 20831 and 20862 cells contained less than the original estimated 60 genome copies. We believe that the copy number of viral genomes at the tandem integration sites is mutable, likely because of recombination events at these loci. A summary of the relative genomic properties of the HPV integration sites in 20861, 20831 and 20862 cells is presented in Supplementary S6C Fig.

In 20831 and 20862 cells, the viral genome was integrated in an intergenic region between the *TP63* and *LEPREL1* genes on chromosome 3, and it appeared that approximately 89 kb of the flanking cellular sequence (chr3: 189,573,774–189,662,800; hg19), which also overlapped with 41.3 kb of the *TP63* gene, had been amplified around 6.6- and 2.5-fold in 20831 and 20862 cells, respectively. RNA-seq identified a viral-cellular fusion transcript of approximately 1.5 kb transcribed from the integration locus in both W12 sub-clones (Supplementary S7A Fig), the expression of which was approximately 2.2-fold (20831 cells) and 6.6-fold (20862 cells) lower than the fusion transcript expressed from the 20861 Type III integration site (Fig 4).

Alignment of ChIP-seq reads to the sequences flanking the integration sites in 20831 and 20862 cells showed similar levels of Brd4 binding and H3K27ac modification to those observed in the same region of 20861 and 20863 cells, which do not have HPV integrated in this region (Supplementary S7B Fig). Similarly, there was not a strong enrichment of super-enhancer markers across the integration site in 20831 and 20862 cells when signals were aligned to our custom reference genome (Supplementary S7C Fig). It should also be noted that the higher copy number of viral genomes relative to the amplified cellular flanking sequence is the result of additional ChIP-seq signals that originate from the Type II integration sites that also exist in these two sub-clones (Supplementary S4 and S5 Figs). In conclusion, the formation of an HPV induced super-enhancer at the site of integration is not a universal event.

### The 20861 integration event hijacked and activated a basal cellular enhancer

**Fig 7:**
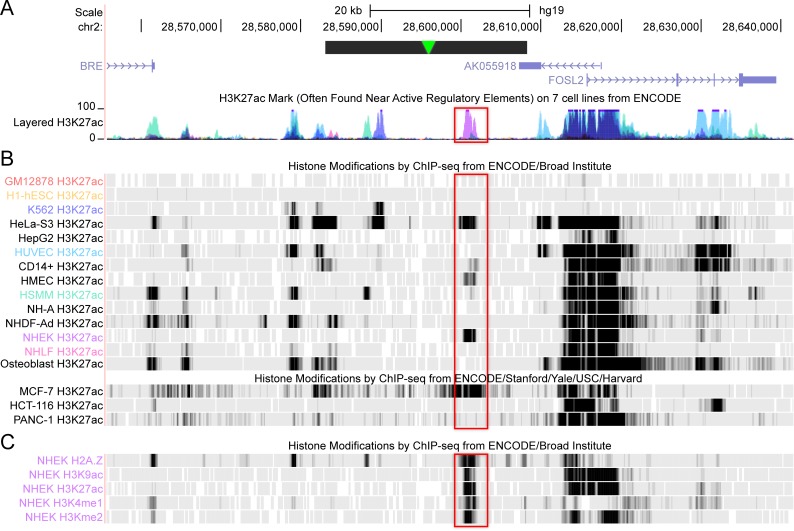
A cellular epithelial-specific enhancer is amplified in the 20861 super-enhancer. **A,** The strong cellular H3K27ac peak shown in Fig 5A aligned with an epithelial-specific enhancer (boxed region) identified from ENCODE. The data from the seven cell lines are represented as colored peaks in the layered H3K27ac track and are highlighted according to the ChIP-seq profiles from the ENCODE/Broad Institute track. The amplified region is marked by a black horizontal bar; the HPV16 genome insertion point is marked by a green arrowhead. **B,** The H3K27ac ChIP-seq profiles from individual cell types are shown (GM12878, B-lymphocytes; H1-hESC, human embryonic stem cells; K562, human erythroleukemic cell line; HeLa-S3, cervical carcinoma; HepG2, hepatocellular carcinoma; HUVEC, human umbilical vein cells; CD14+, CD14-positive monocytes; HMEC, human mammary epithelial cells; HSMM, human skeletal muscle myoblasts; NH-A, normal human astrocytes; NHDF-Ad, normal human dermal fibroblasts-adult; NHEK, normal human epidermal keratinocytes; NHLF, normal human lung fibroblasts; MCF-7, mammary gland adenocarcinoma; HCT-116, colorectal carcinoma; PANC-1, pancreatic carcinoma). **C,** H2A.Z, H3K9ac, H3K4me1, H3K4me2, and H3K27ac tracks in NHEK cells from the ENCODE/Broad Institute track.

Notably, the high peak of H3K27ac activity over the amplified cellular sequence at the 20861 HPV integration locus overlapped with an epithelial-specific enhancer identified from ENCODE data (Bernstein Lab, Broad Institute; Farnham and Snyder Labs, Stanford University) [31–33], as shown in Fig 7. A small peak of H3K27ac activity at this enhancer region was also observed in the 20863 control cells (Fig 5A), supporting its role as a regulatory element in keratinocytes. Furthermore, the ENCODE data also shows this peak to be enriched in H2A.Z, H3K9ac, H3K4me1, and H3K4me2 in NHEK (normal human epidermal keratinocytes). This indicates that HPV16 integrated adjacent to, and multimerized, a basal cellular enhancer, which subsequently synergized with the viral URR enhancer to create a super-enhancer-like element in 20861 cells.

## Discussion

We have recently shown that tandem copies of integrated HPV16 DNA formed a super-enhancer-like element that drives expression of the viral oncogenes in the cervical neoplasia derived cell line, 20861 [22]. In this follow-up study, our aim was to characterize the genetic and epigenetic architecture at this integration locus to better understand the mechanisms that led to the formation of a *de novo* super-enhancer.

Early studies of HPV integration had defined the sites of viral integration as either Type I (a single integrated copy), or Type II (tandem head-to-tail repeats of multiple viral genomes) [8]. Subsequent studies have shown that the flanking cellular sequences are often co-amplified and rearranged [9–12] and so here we designate tandem copies of the HPV genome interspersed with cellular DNA as Type III integrants. WGS of 20861 DNA showed that the viral genome was integrated into chromosome 2 (chr2:28,595,730–28,595,741) and had been amplified about 26-fold along with 25 kb of flanking cellular DNA, forming a Type III viral-host concatemer. This indicates that co-amplification of viral and host DNA occurred after the initial integration event. The initial amplification of virus-host sequences could happen if the viral E1 and E2 replication proteins were still expressed from intact genomes and could initiate DNA synthesis from the integrated viral origin. This could result in onion skin replication and/or rolling circle type replication, which could give rise to viral-host concatamers [9, 10, 34, 35]. Homologous recombination could result in further multimerization of the integration locus [9, 36]. The Type III viral-host concatemer in 20861 cells was further confirmed using fiber-FISH analysis of the integration site with DNA probes against HPV16 and the amplified cellular flanking sequence. This powerful technique allowed us to verify and visualize the repeating tandem units of viral DNA and interspersing cellular DNA at the molecular level.

The transcriptional and epigenetic landscape of the 20861 Type III integration site was analyzed using next-generation sequencing techniques. We initially identified a viral-host fusion transcript by APOT and, as is commonly found, the HPV16 splice donor at nt 880 was joined to a cryptic splice acceptor in the adjacent cellular DNA. RNA-seq analysis confirmed the use of this viral-host splice site, but also identified longer transcripts derived from this locus. This is all consistent with previous RNA analysis of 20861 cells using Northern blots and S1 nuclease mapping analyses [17].

ChIP-seq analysis showed two strong peaks of enrichment of the super-enhancer markers Brd4 and H3K27ac at both the viral URR and adjacent cellular sequence at the integration locus, the latter of which was not observed in the same region of cellular DNA in the W12 20863 sub-clone that harbors extrachromosomal viral DNA. The strong peak of H3K27ac modification within the amplified cellular flanking sequence overlapped with an epithelial-specific enhancer (also defined by H3K27ac) in the ENCODE datasets [31–33]. The identified enhancer was shown to be particularly active in normal human epidermal keratinocytes (NHEK), as well as HeLa cervical carcinoma and MCF-7 mammary gland adenocarcinoma cells, suggesting that it may play a role in regulating pathways relevant to epithelial cell function and/or cancer. We postulate that it is synergy between this enhancer element and the viral URR that led to the development of a super-enhancer-like element that drives high-level expression of the viral *E6* and *E7* oncogenes. We suspect that any enhancer that is active, or could be activated, could be hijacked and amplified and under the correct circumstances develop into a super-enhancer.

HPV integration can occur randomly throughout the entire human genome, but has a tendency towards transcriptionally active regions [6, 7]. Integration may also occur in transcriptionally inactive regions, but it is likely that these do not promote strong expression of E6 and E7 leading to the outgrowth of a clonal population. Integration into transcriptionally active regions could enhance viral gene expression in general, but the hijacking of an adjacent enhancer element could further promote viral oncogene expression. We propose that in 20861 cells, the virus integrated adjacent to, and hijacked, a basal tissue-specific enhancer. Subsequent co-amplification of the viral genome and surrounding cellular sequences resulted in the development of a viral-cellular super-enhancer-like element to strongly drive oncogenic progression.

To show that the development of the 20861 super-enhancer was most likely due to synergy between the viral URR and the adjacent cellular enhancer, we examined two other W12 sub-clones, 20831 and 20862, that were originally described to have ~60 tandem copies of integrated HPV16 DNA. These clones did not show any enrichment of the super-enhancer marker Brd4 at the integration locus by immunofluorescence. Furthermore, ChIP-seq signals for super-enhancer markers at the 20831/20862 integration site were similar to patterns observed in 20861 and 20863 cells, which do not contain integrated HPV at this locus. Correspondingly, these clones had previously been reported to express lower levels of E6/E7 mRNA [8, 17]. We discovered that these clones were related to each other as they both contained HPV16 integrated into the same site in chromosome 3, which is very consistent with earlier analyses [8, 17]. However, we noted that the number of viral genomes were less than originally reported, and our preliminary observations suggest that the copy numbers at each integration site are unstable/dynamic. Focal amplification was significantly greater at the Type III integration site in 20861 cells relative to the other integrated W12 sub-clones, which may be the result of increased genomic instability at the locus due to higher *E6/E7* oncogene expression. We also examined an additional six cervical carcinoma derived cell lines (C-33A, C-411, CasKi, HeLa, ME-180 and SiHa) for a prominent Brd4 focus but none was apparent. Therefore, the development of a strong super-enhancer at the site of HPV integration is not universal and likely results from a combination of factors.

The location of HPV insertional breakpoints within the human genome can influence the transcription of neighboring genes [10, 20, 37] and HPV integration into, or adjacent to, cancer-associated genes has been reported to be a potential mechanism for HPV-mediated oncogenic progression in some cases [11, 19, 38]. Altered transcription can be restricted to the disrupted gene with minimal or no effects on neighboring transcripts [20], or can extend to contiguous genes [10, 20]. However, in almost all cases oncogenesis is primarily driven by, and dependent on, the *E6* and *E7* viral oncogenes.

At the 20861 Type III integration locus, the HPV16 genome integrated into an intergenic region on chromosome 2, and the subsequent focal amplification of the surrounding cellular sequence overlapped with a non-coding RNA located downstream of the integration site. Integration also resulted in upregulation of *FOSL2* (3.5-fold, adjusted p-value 9.2 x 10^−6^), which is located approximately 20 kb downstream of the HPV integration site, just outside the amplified region (GEO accession number GSE75987) [22]. Therefore, in addition to driving high *E6/E7* expression, the super-enhancer increased transcription of neighboring genes. We previously showed that disruption of Brd4 from the 20861 super-enhancer resulted in down-regulation of *E6* and *E7* gene expression and induction of cellular senescence [22]. However, *FOSL2* levels were only minimally reduced, indicating that 20861 cell proliferation is completely dependent on expression of the *E6* and *E7* oncogenes.

Southern blot analysis of the W12 cell clones had indicated that they contained both unit length genomes (most likely head-to-tail copies of the viral DNA; Type II integration) as well as amplified viral-cellular junctional DNA (Type III integration) [8]. The fiber-FISH experiments presented here confirmed that the clones examined all contained at least two independent integration events. Each clone examined contained a Type II and a Type III integration site located at different regions of the host genome. Although each clone contains Type II and Type III integration sites, our data suggests that only the Type III integration site is transcriptionally active. RNA-seq analysis detected only transcripts from the Type III viral-host junctional sequences in all three clones. We confirmed this by absolutely quantifying the amount of mRNA containing the viral E6*I splice with that of the downstream cellular sequences (Supplementary S6E–S6G Fig). This is in line with previous studies, which reported that fusion mRNAs were expressed from a single integration site in tumors containing multiple integration loci [39, 40]. This is also consistent with initial studies that showed that no RNA sequences from the 3’ end of the HPV16 early region could be detected in 20861, 20831 and 20862 cells [8, 17]. Distinct from 20861 cells, the 20831/20862 fusion transcript contained the HPV16 E1^E4 splice (nt 880^3358) before running into the cellular sequence at nt 3741. It has been shown that viral sequences from the HPV16 3’ early region decrease the steady-state expression, stability and translation of early mRNAs [17, 41] and replacement of these sequences with host sequences can increase expression of *E6* and *E7*.

In normal cells, repetitive DNA sequences are silenced through methylation to prevent genomic instability at these regions [42]. Kalantari et al. have previously shown that tandemly integrated viral DNA is hypermethylated in CasKi, 20831 and 20862 cells, whereas viral DNA is hypomethylated in 20861 cells [43]. We show here that the majority of viral genome copies are located in the transcriptionally active Type III integration locus in 20861 cells, while in 20831/20862 cells most viral genomes are in the Type II integration locus. It is possible that the head-to-tail tandem copies of the viral genome at Type II integration sites are recognized and silenced through spreading methylation [44], whereas Type III integration events can escape such epigenetic silencing due to the intervening cellular sequences.

There are several ways in which integration of the HPV genome into the host can deregulate expression of the *E6* and *E7* oncogenes and promote oncogenesis [13]. Here, we describe a new way in which the virus hijacks an adjacent cellular enhancer to drive strong, deregulated expression of E6/E7. Amplification of the viral-host DNA at this site resulted in the formation of a strong super-enhancer-like element. Thus, there is no universal mechanism by which integration of an oncogenic HPV promotes oncogenesis; instead it depends on many stochastic factors such as viral copy number, associated amplification and rearrangements, the existence of a convenient host splice acceptor and/or polyadenylation site, and the epigenetic signature of the locus.

## Materials and methods

### Cell culture

W12-derived subclones [8, 17] and NIKS human keratinocyte [45] cell lines were maintained in F-medium (3:1 [vol/vol] F-12–Dulbecco’s modified Eagle’s medium, 5% fetal bovine serum, 0.4 μg/ml hydrocortisone, 5 μg/ml insulin, 8.4 ng/ml cholera toxin, 10 ng/ml epidermal growth factor, 24 μg/ml adenine, 100 U/ml penicillin and 100 μg/ml streptomycin). All cells were grown in the presence of irradiated 3T3-J2 feeder cells. C-33A, C-411, CasKi, HeLa, ME-180 and SiHa cervical carcinoma derived cell lines were maintained in Dulbecco’s modified Eagle’s medium, 10% fetal bovine serum, 100 U/ml penicillin and 100 μg/ml streptomycin).

### Amplification of Papillomavirus Oncogene Transcripts (APOT)

APOT was performed as described previously [27]. See Supplementary S2 Table for primer sequences. The resulting PCR product band was gel purified using Roche High Pure PCR Product Purification Kit and sequenced. The cellular DNA fused to HPV16 was identified using BLAT Search on the UCSC Genome Browser.

### Southern blot analysis

Total DNA was harvested with the DNeasy Blood and Tissue kit (Qiagen). 1 μg total DNA was digested with either a single-cut linearizing enzyme (BamHI) for the HPV16 genome or a non-cutter (HindIII) to linearize cellular DNA. After digestion, samples were separated on 0.8% Tris-acetate-EDTA (TAE) agarose gels. A known quantity of linear HPV16 DNA was run on the same gel as the samples. DNA was visualized with 0.5 mg/ml ethidium bromide and transferred onto nylon membranes with a TurboBlotter downward transfer system (Whatman). Membranes were UV cross-linked, dried, incubated with prehybridization blocking buffer (3× SSC [1× SSC is 0.15 M NaCl plus 0.015 M sodium citrate], 2% SDS, 5× Denhardt’s solution, 0.2 mg/ml sonicated salmon sperm DNA), and then incubated overnight with [^32^P]-dCTP-labeled HPV16 DNA in hybridization buffer (3× SSC [1× SSC is 0.15 M NaCl plus 0.015 M sodium citrate], 2% SDS, 5× Denhardt’s solution, 0.02 mg/ml sonicated salmon sperm DNA, 25 ng [^32^P]-dCTP-labeled HPV16 DNA). Radiolabeled probe was generated from 50 ng of gel-purified linear HPV16 DNA with a Random Prime DNA labeling kit (Roche). Hybridized DNA was visualized and quantitated by phosphorimaging on a Typhoon Scanner (GE Bioscience).

### Whole genome sequencing (WGS)

Total cellular DNA was purified from 2 x 10^6^ W12 cells using the DNeasy Blood and Tissue kit (Qiagen) and sequenced (2 x 150 bp paired-end reads) using the Illumina HiSeq 4000 platform (Genomics Resource Center, Institute for Genome Sciences, University of Maryland). Alignment of WGS data was performed using Bowtie 2 (version 2.2.5) [46, 47].

### Custom viral genome hybrid capture sequencing

To confirm virus-host insertional breakpoints, we designed a custom panel of Agilent SureSelect XT v.G.1 HPV16 genome sequences to detect viral genomic sequences and flanking host genomic DNA sequences in various samples (manuscript in preparation, Symer et al., similar in overall approach to [48]). We performed the enrichment and library preparation using a protocol on the Agilent Bravo following the manufacturer’s protocol in the Genomics Shared Resource, Ohio State University Comprehensive Cancer Center. Libraries were sequenced using a HiSeq 2500 (2 x 150-bp paired-end reads) at Nationwide Children’s Hospital. More than 5 million reads were sequenced per sample (i.e. for cell line 20861, we generated 5.5 million reads, and for 20863, 23 million reads; see Supplementary S1 Table). Reads were aligned against a custom reference genome assembly (i.e. human genome sequence (UCSC hg19 genome) + HPV16 genome) using BWA aligner [49]. Discordant sequence read pairs having one end aligned to the HPV16 viral genome and the other end aligned to the human genome were re-aligned using the GSNAP aligner [50]. After removing the duplicate pairs from these reads, we used a custom pipeline involving Hydra [10, 51] to identify and confirm HPV insertional breakpoints in the human genome. Many of the breakpoints observed in chromosome 2 in the 20863 cell line were located at simple repeat regions, so those calls were supported by low numbers of paired end reads and were made at low confidence (Supplementary S1 Table).

### Immunofluorescence (IF)

Cells were cultured on coverslips and fixed at room temperature in 4% paraformaldehyde (PFA)–PBS for 20 minutes. Cells were permeabilized in 0.1% Triton X-100 and stained with primary and fluorescent secondary antibodies using standard procedures. Coverslips were mounted in ProLong Gold containing 4’,6-diamidino-2-phenylindole (DAPI) for analysis by confocal microscopy. Anti-BRD4 CW152 rabbit polyclonal antibody was used at 1:100 dilution [52].

### Fluorescent *in situ* hybridization (FISH)

Cells grown on coverslips were fixed in cold methanol-acetic acid (3:1) for 1–3 minutes followed by 4% paraformaldehyde–PBS for 10 minutes. Cells were treated with RNase A for 1 hour at 37°C and dehydrated in a 70%, 90%, and 100% ethanol series for 3 minutes each. Green 5-Fluorescein dUTP labelled BAC clone RP11-1140H22 (chr2: 28,504,596–28,660,966; hg19) was purchased from Empire Genomics. DNA-FISH probe against full-length HPV16 DNA was fluorescently labeled using the Alexa Fluor-594 FISH Tag DNA Multicolor Kit (Life Technologies) following manufacturer’s protocol. To each coverslip, 40–50 ng labeled DNA FISH-probe in hybridization buffer (Empire Genomics) supplemented with 50 μg/ml human Cot-1 DNA (Invitrogen) was added. DNA denaturation was performed at 75°C for 5 minutes, followed by hybridization at 37°C overnight. Cells were washed for 5 minutes each with 1x phosphate-buffered detergent (Qbiogene) at room temperature, 1x wash buffer (0.5x SSC, 0.1% SDS) at 65°C, and 1x phosphate-buffered detergent at room temperature. Coverslips were mounted in ProLong Gold (ThermoFisher) containing DAPI for analysis by confocal microscopy.

### IF-FISH

Cells grown on coverslips were fixed in cold methanol-acetic acid (3:1) for 1–2 minutes followed by 4% paraformaldehyde–PBS for 10 minutes. Cells were processed for immunofluorescence staining followed by DNA FISH using methods described above and as described previously [53].

### ChIP-qPCR

W12 cells were processed as previously described [22]. Briefly, 5 x 10^7^ cells were crossed-linked with 1% formaldehyde and sheared to 100–500 bp DNA fragments using a Bioruptor sonicator (Diagonode) on high power settings. An aliquot of the lysate (5%) was processed for input control. Chromatin samples (20 μg per ChIP) were incubated overnight at 4°C with antibodies against Brd4 (Bethyl Laboratories A301-985A, 3 μg) and H3K27ac (Millipore 07–360, 3 μl). No-antibody controls were included to measure non-specific binding. Chromatin-immunocomplexes were precipitated for 1 hour at 4°C with blocked Dynabeads Protein G (Invitrogen), subjected to multiple wash steps and the chromatin eluted. Recovered DNA was reverse cross-linked overnight at 65°C with 5 M NaCl, followed by RNase A and proteinase K treatment, and purified using the ChIP DNA Clean & Concentrator kit (Zymo Research). ChIP DNA was analyzed by real-time qPCR, using primers listed in Supplementary S2 Table. Real-time qPCR was performed using the ABI Prism 7900HT sequence detector (Applied Biosystems) and SYBR green PCR master mix (Applied Biosystems). All reactions were run in triplicate and compared to standard curves of input chromatin DNA or cloned HPV16 genome from W12 cells, for determination of viral copy number, to generate a standard curve of threshold cycle (CT) versus log10 quantity (fg).

### ChIP-seq

W12 cells were processed as described above for ChIP-qPCR. ChIP DNA samples were pooled and sequenced (2 x 150 bp paired-end reads) using the Illumina HiSeq 4000 platform (Genomics Resource Center, Institute for Genome Sciences, University of Maryland). Alignment of ChIP-seq data was performed using Bowtie 2 (version 2.2.5) [46, 47].

### RNA-seq

Total RNA was purified from W12 cells using the RNeasy Mini Kit (Qiagen) and RNA integrity determined using the Bioanalyzer 2100 (Agilent Technologies). Polyadenylated RNA was sequenced (2 x 150 bp paired-end reads) using the Illumina HiSeq 4000 platform (Genomics Resource Center, Institute for Genome Sciences, University of Maryland). RNA-seq data was aligned to reference genomes using TopHat2 (version 2.1.0) [54] and expression analysis of known transcripts (hg19_refseq_16_08_01_v2) performed using Cufflinks (version 2.2.1) [55].

### HPV16 biotinylated probe synthesis

HPV16 DNA inserted into the BamHI site of pUC18/19 was digested with BamHI and extracted from a 0.8% agarose gel to remove pUC18/19. Biotinylated HPV16 genome probe was synthesized with BioPrime DNA Labeling System (Invitrogen, 18094011) using 500 ng of digested HPV16 DNA incubated at 37°C for 4 hours. Biotinylated probe was purified using QIAquick PCR Purification Kit (Qiagen, 28104).

### Genomic DNA plug preparation

W12 cells were harvested, pelleted for 5 minutes at 1,000 rpm, and resuspended in cold 1x PBS to a dilution of 1 x 10^6^ cells per ml. Diluted cells were warmed to 37°C for 1–2 minutes, and an equal volume of 1.5% SeaPlaque agarose (Lonza, 50100) in 1x PBS, warmed to 45°C, was added. Cell/agarose mixture was aliquoted into plug mold wells (Biorad, 1703713) and solidified for 20 minutes at 4°C. Solid plugs were ejected into lysis buffer (1 mg/ml proteinase K [added directly before use], 100 mM EDTA, 1% N-lauroylsarcosine, 10 mM Tris-HCl, pH 8.0, and incubated overnight in a 50°C water bath. Lysis buffer was discarded and plugs were rinsed with 1x TE. Rinsed plugs were washed three times for 1 hour in 1x TE. Washed plugs were stored for several months in 1x TE at 4°C.

### DNA combing

The washed plug was transferred to a 2 ml tube and melted for 20 minutes at 70°C in 0.1 M MES (pH 6.5). Melted plug was cooled for 5 minutes in a 42°C water bath. 3 units of β-agarase (New England BioLabs, M0392S) were added to cooled DNA solution and incubated overnight in a 42°C water bath. DNA solution was carefully poured into a 2 ml Teflon reservoir. A silanized coverslip was inserted into the DNA solution and incubated for 5 minutes. The coverslip was removed and adhered to a glass slide with cyanoacrylate glue. A test coverslip was pulled, stained with YOYO-1 (2:10,000 YOYO-1 in 0.1 M MES), and visualized with a microscope to ensure quality and density of DNA fibers. Coverslips were baked for 30 minutes at 60°C to crosslink DNA fibers to coverslip.

### Fiber-FISH

DNA fibers were denatured for 20 minutes in 0.2 M NaOH. Slides were neutralized 5 times with 1x PBS (pH 7.4) for 1 minute. Slides were dehydrated for 3 minutes each in successive baths of 70%, 90%, and 100% EtOH and air dried for 8 minutes. 500 ng of HPV16 biotinylated probe was mixed with 5 μg of human Cot-1 DNA and hybridization buffer (50% formamide, 2x SSC, 0.5% SDS, 0.5% N-lauroylsarcosine sodium salt, 1% Blocking reagent [Roche, 11096176001], 10 mM NaCl) up to a total volume of 20 μL. Probe mixture was added to coverslip, a clean coverslip was placed on top and sealed with rubber cement. Slides were denatured for 5 minutes at 75°C and then incubated for 16 hours at 37°C. The rubber cement and top coverslips were removed from the slides and the slides rinsed with 2x SSC. The slides were washed three times with 2x SSC/50% formamide for 5 minutes, and then washed three times with 2x SSC for 5 minutes. Slides were blocked for 1 hour in a humidified chamber with 5% BSA in 1x PBS and washed 3 times for 5 minutes with PBS-T (0.1% Triton X-100 in 1x PBS). To visualize biotinylated probe, slides were incubated in a humidified chamber at 37°C in the following layers. Layer 1: 1:100 streptavidin, Alexa Fluor-488 conjugated (ThermoFisher, S11223) in 5% BSA/1x PBS for 30 minutes. Layer 2: 1:200 biotinylated anti-streptavidin (Vector, BA-0500) in 5% BSA/1x PBS for 1 hour. Layer 3: 1:200 streptavidin, Alexa Fluor-488 conjugated in 5% BSA/1x PBS for 30 minutes. Layer 4: 1:200 biotinylated anti-streptavidin and 1:200 anti-ssDNA IgG2a (EMD Millipore, MAB3034) in 5% BSA/1x PBS for 1 hour. Layer 5: 1:200 streptavidin, Alexa Fluor-488 conjugated, and 1:100 goat anti-mouse IgG2a, Alexa Fluor-647 conjugated (Invitrogen, A-21241), in 5% BSA/1x PBS. Three 5 minute washes with PBS-T were performed in between each layer. Slides were rinsed with 1x PBS, mounted with ProLong Gold (ThermoFisher, P36930).

### Two color fiber-FISH

Digoxigenin labeled probes were synthesized with BioPrime DNA Labeling System (Invitrogen, 18094011) using 500 ng BsaBI and XcmI digested HPV16 DNA. The biotinylated nucleotide mix from the kit was replaced with PCR DIG Probe Synthesis Mix (10x, PCR DIG Probe Synthesis Kit, Roche, 11636090910) and the labeling reaction incubated at 37°C for 4 hours. The DIG probe was purified using QIAquick PCR Purification Kit (Qiagen, 28104). Biotinylated probes were prepared as described previously. Following combing, DNA fibers were denatured for 20 minutes in 0.2 M NaOH, and neutralized five times with 1x PBS (pH 7.4) for 1 minute. Slides were dehydrated for 3 minutes each in successive baths of 70%, 90%, and 100% EtOH and air dried for 8 minutes. Biotinylated/DIG probes in an equivalent ng/kb ratio, 200 ng of an 8 kb probe and 620 ng of a 25 kb probe, were mixed with 5 μg human Cot-1 DNA in hybridization buffer (50% formamide, 2x SSC, 0.5% SDS, 0.5% N-lauroylsarcosine sodium salt, 1% Blocking reagent [Roche, 11096176001], 10 mM NaCl) to a total volume of 20 μL. Probe mixture was added to the coverslip, a clean coverslip was placed on top and sealed with rubber cement. Slides were denatured for 5 minutes at 75°C and incubated for 16 hours at 37°C. Slides were washed three times with 2x SSC/50% formamide for 5 minutes and three times with 2x SSC for 5 minutes. Slides were blocked for 1 hour in a humidified chamber with 5% BSA in 1x PBS and washed 3 times for 5 minutes with PBS-T (0.1% Triton X-100 in 1x PBS). To visualize the biotin and DIG-labeled probes, slides were incubated in a humidified chamber at 37°C with the following layers: Layer 1: 1:100 streptavidin, Alexa Fluor-555 conjugated (ThermoFisher, S21381), and 1:100 anti-digoxigenin, FITC conjugated (Roch, 11333089001), in 5% BSA/1x PBS for 1 hour. Layer 2: 1:200 biotinylated anti-streptavidin (Vector, BA-0500) and 1:200 anti-FITC (Invitrogen, 711900) in 5% BSA/1x PBS for 45 minutes. Layer 3: 1:200 streptavidin, Alexa Fluor-555 conjugated in 5% BSA/1x PBS for 30 minutes. Layer 4: 1:200 biotinylated anti-streptavidin, 1:200 anti-rabbit, FITC conjugated (Rockland, 611-102-122), and 1:200 anti-ssDNA IgG2a (EMD Millipore, MAB3034) in 5% BSA/1x PBS for 45 minutes. Layer 5: 1:200 streptavidin, Alexa Fluor-555 conjugated, and 1:100 goat anti-mouse IgG2a, Alexa Fluor-647 conjugated (Invitrogen, A-21241), in 5% BSA/1x PBS. Three 5 minute washes with PBS-T were performed in between each layer. Slides were rinsed with 1x PBS, mounted with ProLong Gold (ThermoFisher, P36930). All slides were imaged using a Leica TCS-SP5 laser scanning confocal imaging system.

### Fiber measurements

Measurement of signal lengths from raw Leica files was performed using Bitplane Imaris x64. Images were analyzed in Surpass with an orthogonal camera. Measurement points of equal diameter were placed on the first and last points of signal for each HPV16 genome. Dots of signal were considered contiguous if they were within 0.6 μm of a previous dot. Genomes continuous in a fiber were measured with a single line of measurement points to determine the length of any interspersing DNA. Base pair length was determined using 1 μm = 2 kb conversion.

## Supporting information

S1 Fig Type II fiber-FISH in 20861 cells. **A,** Representative image of a fiber containing the 20861 type II integration site. The DNA strand was visualized with a single stranded DNA antibody, shown in red. The HPV16 FISH signal is shown in green. **B,** Scatter plot showing number of HPV16 genomes per individual DNA fiber. Average signal count per fiber and SD is shown along with total number of fiber counts. Greatest number of genomes found in a single fiber is 16.4. HPV16 genome copy number was calculated by dividing site length (kb) by 7.9 kb (unit length of HPV16 genome). Data are from two biological replicates.
(EPS)

S2 Fig Alignment of WGS data for the W12 sub-clones to the HPV16 genome. Alignment of WGS data from the 20861, 20862, 20831 and 20863 cell lines to the HPV16 isolate 16W12E (AF125673.1) reference genome. Histograms represent copy numbers of viral sequences (blue) and counts of discordant paired-end reads supporting insertional (red) breakpoints. The scale (y-axis) of each plot was normalized to maximum read counts. HPV breakpoints are defined further in Table 1 and S1 Table. The alignments for 20861 and 20863 are duplicated from Fig 2C for reference.
(EPS)

S3 Fig Prominent Brd4 focus observed in 20861 cells is not characteristic of other HPV-positive cells. Brd4 was detected in NIKS (HPV-negative), W12 sub-clones (20861, 20831 and 20862) and cervical carcinoma derived cell lines (C-33A (HPV-negative), C-411, CasKi, HeLa, ME-180 and SiHa) by indirect immunofluorescence with the CW152 Brd4 antibody (green). Data and images shown were obtained from z-stacks of the entire cell combined using maximum projection. DAPI was used as a nuclear stain.
(PDF)

S4 Fig Type II and III fiber-FISH in 20831 cells. **A,** Representative image of a fiber containing the 20831 Type III integration site. Image is representative of a conserved pattern. The DNA backbone was visualized with a single stranded DNA antibody, shown in red. The Type III site of 20831 cells contains two sizes of integrated HPV16, whole and partial, and three sizes of interspersing host DNA, large, small, and smallest. **B,** Scatter plot showing number of HPV16 genomes per individual DNA fiber in the Type III integration site. Average signal count per fiber and SD is shown along with total number of counts. Data are from two biological replicates. Greatest number of genomes found in a single fiber is 18. **C,** Scatter plot showing measured HPV16 signal lengths. Two populations of HPV16 signal size represent whole (N = 240) and partial (N = 72) genomes shown in **A**. Data are from two biological replicates. **D,** Scatter plot showing measured length of the interspersing space between HPV16 signals from every fiber. Three populations of interspersing host DNA lengths represent the large (N = 96), small (N = 85), and smallest (N = 69) lengths seen in **A**. Measurements were based on the conversion 1 μm = 2 kb. Average measured length in kb and SD is shown along with total number of fibers. Data are from two biological replicates. **E,** Representative image of a fiber containing the 20831 Type II integration site. The DNA backbone was visualized with a single stranded DNA antibody, shown in red. **F,** Scatter plot showing number of HPV16 genomes per individual DNA fiber of the Type II integration site. Average signal count per fiber and SD is shown along with total number of fibers. Greatest number of genomes found in a single fiber is 56.7. HPV16 genome number was calculated by dividing site length (kb) by 7.9 kb (unit length of HPV16 genome). Data are from two biological replicates.
(EPS)

S5 Fig Type II and III fiber-FISH in 20862 cells. **A,** Representative image of a fiber containing the 20862 Type III integration site. The DNA backbone was visualized with a single stranded DNA antibody, shown in red. **B,** Scatter plot showing number of HPV16 genomes per individual DNA fiber of the Type III integration site. Average signal count per fiber and SD is shown along with total number of counts. Greatest number of genomes found in a single fiber is 13. Data are from two biological replicates. **C,** Scatter plot showing measured HPV16 signal length for the Type III integration site. **D,** Scatter plot showing measured length of the interspersing space between HPV16 signals from every fiber. Two populations of differing interspersing host length units could represent separate patterns of Type III integration in 20862 cells. Measurements were based on the conversion 1 μm = 2 kb. Average measured length in kb and SD is shown along with total number of fibers. **E,** Representative image of a fiber containing the 20862 Type II integration site. The DNA backbone was visualized with a single stranded DNA antibody, shown in red. **F,** Scatter plot showing number of HPV16 genomes per individual DNA fiber of the Type II integration site. Average signal count per fiber and SD is shown along with total number of fibers. Greatest number of genomes found in a single fiber is 59.8. HPV16 genome number was calculated by dividing site length (kb) by 7.9 kb (unit length of HPV16 genome). Data are from two biological replicates.
(EPS)

S6 Fig Analysis of 20831 and 20861 W12 clones. **A,** Southern blot analysis of W12 cell lines. Genomic DNA was digested with either HindIII (H; does not cut HPV16 DNA) or BamH1 (B; a single cut in HPV16 DNA). DNA fragments were separated by electrophoresis, transferred to membranes and probed with ^32^P-labeled HPV16 DNA. Arrows indicate Type II or Type II integration patterns. Southern blot is duplicated from Fig 1A to include all W12 subclones analyzed. **B,** Predicted BamHI and HindIII restriction fragments at the 20861, 20831 and 20862 Type III integration loci. **C,** Summary of properties of the 20861, 20831 and 20862 Type III integration loci. ^a^ Determined from Southern blot analysis (see A and B); ^b^ Fold difference in Type III fusion mRNA expression relative to 20861 cells; ^c^ With respect to transcriptionally active Type III integration site, determined by ChIP-seq and immunofluorescence. **D,** Total DNA was extracted from 20861 and 20863 cells and qPCR analysis performed using primers targeting the cellular flanking sequence at the 20861 integration site. Copy number was determined against a standard curve and normalized to *ACTB*. Average copy number was calculated from two independent experiments. Error bars represent SD. See Supplementary S2 Table for primer sequences. **E-F,** Total RNA was extracted from 20831, 20861 and 20862 cells and RT-qPCR performed using primers targeting E6*I splice transcript and viral-host fusion mRNAs. mRNA expression levels were calculated against a standard curve and normalized to *PPIA*. Error bars represent SD across three independent experiments. See Supplementary S2 Table for primer sequences.
(EPS)

S7 Fig HPV-mediated super-enhancer formation does not occur at all sites of tandem HPV integration. **A,** Alignment of RNA-seq reads from W12 20831 and 20862 cells to the human (hg19) and HPV type 16 isolate 16W12E (AF125673.1) reference genomes showed expression of a viral-cellular fusion transcript spliced into a cryptic acceptor within an intergenic region at chr3: 189,662,800. Data are averaged from three biological replicates. **B,** Alignment of ChIP-seq reads to the human reference genome (hg19) in 20863 cells containing extrachromosomal HPV16, and 20861, 20831 and 20862 cells containing integrated HPV16 genomes. Brd4 and H3K27ac signatures encompassing the integration locus at chr3: 189,662,800 are similar across all four W12 sub-clones. Data are averaged from two biological replicates. **C,** Alignment of ChIP-seq reads to human and HPV type 16 isolate 16W12E (AF125673.1) reference genomes in 20831 and 20862 cells did not show enrichment of super-enhancer markers over the Type III integration site as is the case in 20861 cells (see Fig 5). Enrichment of reads over the viral genome is indicative of signal at Type II integration sites which account for a higher proportion of the viral copy numbers within these two subclones (see S4 and S5 Figs). Histograms represent depth of coverage of aligned reads. Amplified region is marked by a black horizontal bar; HPV16 genome and cellular genes are represented by green and blue horizontal arrows, respectively. Data are averaged from two biological replicates.
(EPS)

S1 Table HPV insertional breakpoints in 20861 and 20863 cells, detected by custom viral genome hybrid capture sequencing. Break IDs were assigned arbitrarily by Hydra insertional breakpoint detection software. Positions of neighboring genes were defined by the orientation of the most proximal integrated HPV16 genome. *Bold font;* the most strongly supported insertional breakpoints matched analysis of WGS data (see Table 1). We used hydra protocol to detect HPV breakpoints from paired-end sequencing data. We show breakpoints supported by at least four discordant read pairs (i.e. one end aligned to HPV and the other end aligned to human genome). We annotated the nearest three (or less) genes located within 300 kb from the breakpoints, using the NCBI RefSeq database. All human genome coordinates are based on UCSC hg19 genome assembly.
(PDF)

S2 Table Primer sequences used for APOT assay, ChIP-qPCR and generation of FISH probe. (PDF)
