# Supplementary figures and images for: HPV integration hijacks and multimerizes a cellular enhancer to generate a viral-cellular super-enhancer that drives high viral oncogene expression
Source: PLoS Genet. 2018 Jan 24;14(1):e1007179. doi: 10.1371/journal.pgen.1007179 (PMC5798845; doi:10.1371/journal.pgen.1007179)

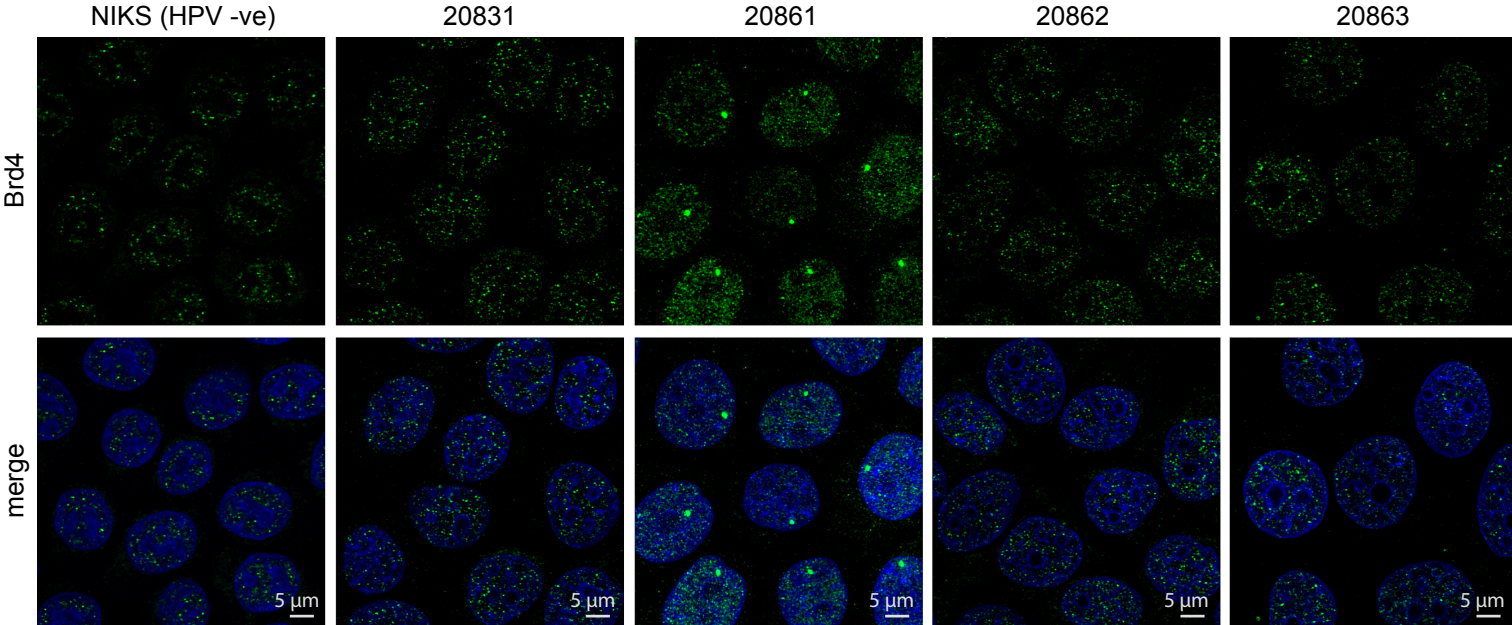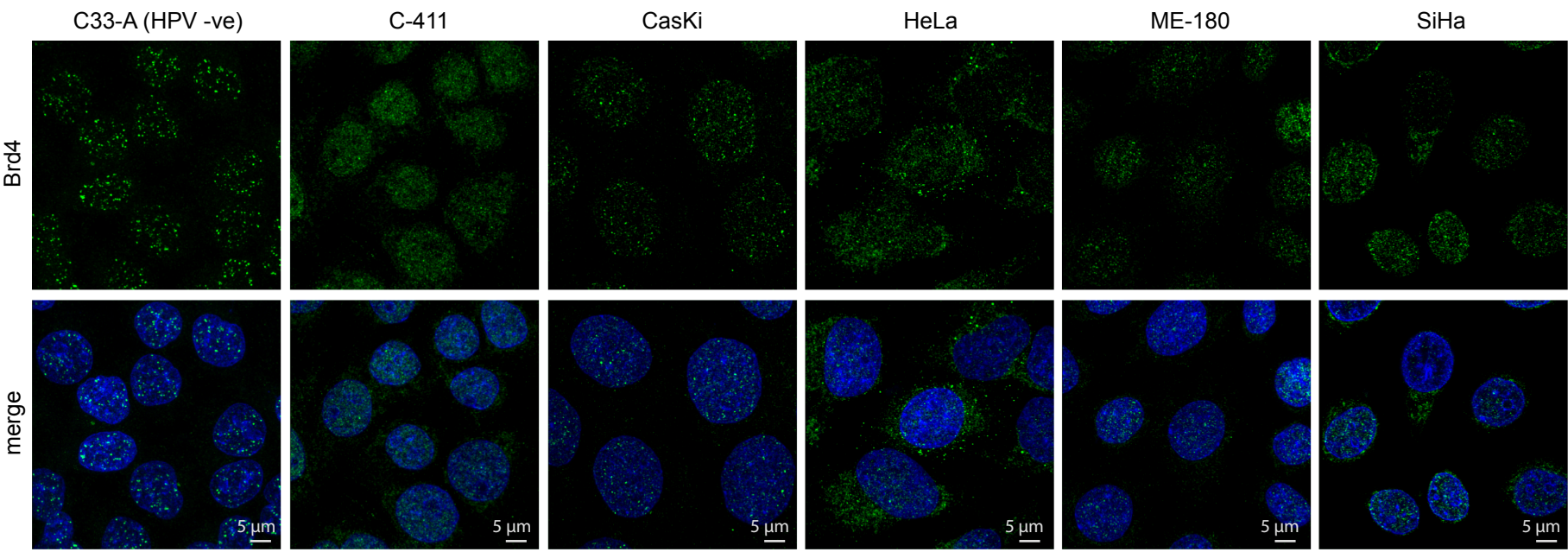

Supplement: S3 Fig — Brd4 was detected in NIKS (HPV-negative), W12 sub-clones (20861, 20831 and 20862) and cervical carcinoma derived cell lines (C-33A (HPV-negative), C-411, CasKi, HeLa, ME-180 and SiHa) by indirect immunofluorescence with the CW152 Brd4 antibody (green). Data and images shown were obtained from z-stacks of the entire cell combined using maximum projection. DAPI was used as a nuclear stain. (PDF) [file pgen.1007179.s003.pdf]
