# Supplementary material for: HPV integration hijacks and multimerizes a cellular enhancer to generate a viral-cellular super-enhancer that drives high viral oncogene expression
Source: PLoS Genet. 2018 Jan 24;14(1):e1007179. doi: 10.1371/journal.pgen.1007179 (PMC5798845; doi:10.1371/journal.pgen.1007179)
Supplement: S1 Table — Break IDs were assigned arbitrarily by Hydra insertional breakpoint detection software. Positions of neighboring genes were defined by the orientation of the most proximal integrated HPV16 genome. Bold font; the most strongly supported insertional breakpoints matched analysis of WGS data (see Table 1). We used hydra protocol to detect HPV breakpoints from paired-end sequencing data. We show breakpoints supported by at least four discordant read pairs (i.e. one end aligned to HPV and the other end aligned to human genome). We annotated the nearest three (or less) genes located within 300 kb from the breakpoints, using the NCBI RefSeq database. All human genome coordinates are based on UCSC hg19 genome assembly. (PDF) [file pgen.1007179.s008.pdf]

S1 Table. HPV insertional breakpoints in 20861 and 20863 cells, detected by custom viral genome hybrid capture sequencing

Cell line	Break ID	Number of reads	Position (HPV16 genome)	HPV gene	HPV read strand	Position (hg19)	Host genome read strand	Neighboring host genes
20861	6	7	2827-3062	E2	+	chr2: 28595279-28595577	+	AK055918
**20861**	**5**	**338**	**2505-3062**	**E1/E2**	**+**	**chr2: 28595311-28595730**	**+**	**AK055918**
20861	7	4	2173-2575	E1	+	chr2: 28595585-28595729	+	AK055918 (downstream 11.546 kb); FOSL2 (upstream 20.049 kb); BRE (downstream 33.818 kb)
**20861**	**10**	**616**	**3151-3678**	**E2/E4**	**-**	**chr2: 28595716-28596131**	**-**	**AK055918 (downstream 11.144 kb); FOSL2 (upstream 19.647 kb); BRE (downstream 33.949 kb)**
20861	12	4	3693-4038	E2/E5	-	chr2: 28595741-28595968	-	AK055918 (downstream 11.307 kb); FOSL2 (upstream 19.81 kb); BRE (downstream 33.974 kb)
20861	11	12	3151-3435	E2/E4	-	chr2: 28595896-28596474	-	AK055918 (downstream 10.801 kb); FOSL2 (upstream 19.304 kb); BRE (downstream 34.129 kb)
20861	18	4	3398-3727	E2/E4	-	chr2: 33141274-33141664	-	LINC00486; LOC100271832 (upstream 10.529 kb); LTBP1 (upstream 30.704 kb); TTC27 (downstream 95.156 kb)
20861	19	4	2634-2813	E1/E2	-	chr2: 33141297-33141606	-	LINC00486; LOC100271832 (upstream 10.587 kb); LTBP1 (upstream 30.762 kb); TTC27 (downstream 95.179 kb)
20861	20	9	276-1039	E6/E7/E1	-	chr2: 33141300-33141691	-	LINC00486; LOC100271832 (upstream 10.502 kb); LTBP1 (upstream 30.677 kb); TTC27 (downstream 95.182 kb)
20861	14	4	6649-7300	L1/URR	-	chr2: 33141421-33141651	-	LINC00486; LOC100271832 (upstream 10.542 kb); LTBP1 (upstream 30.717 kb); TTC27 (downstream 95.303 kb)
20861	16	5	4258-4862	L2	-	chr2: 33141427-33141690	-	LINC00486; LOC100271832 (upstream 10.503 kb); LTBP1 (upstream 30.678 kb); TTC27 (downstream 95.309 kb)
20861	26	8	2905-3288	E2	-	chr7: 124000000-124000000	+	TMEM229A (upstream 52.477 kb); SPAM1 (downstream 114.539 kb)
20861	24	12	2831-3093	E2	+	chr7: 125000000-125000000	+	LOC101928283 (downstream 67.402 kb); LOC101928254 (upstream 182.432 kb)
20861	28	9	2565-2976	E1/E2	-	chr7: 125000000-125000000	-	LOC101928283 (downstream 76.519 kb); LOC101928254 (upstream 191.549 kb)
20861	2	4	4212-4418	L2	-	chr12: 80007755-80007807	+	PAWR; MIR5692B (downstream 155.315 kb); PPP1R12A (downstream 159.535 kb); SYT1 (downstream 161.967 kb); MIR1252 (downstream 194.654 kb)
20861	22	5	4173-4408	L2	-	chr20: 52118023-52118075	-	TSHZ2 (downstream 6.154 kb); LOC101927770 (upstream 51.233 kb)
20863	3	5	4177-4385	L2	-	chr1: 22381406-22381464	+	CDC42; LINC00339 (downstream 23.689 kb); LOC101928043 (upstream 28.865 kb)
20863	7	4	4177-4633	L2	-	chr1: 199440186-199440241	-	LINC01222 (upstream 452.093 kb)
20863	38	12	578-1306	E7/E1	-	chr2: 33141280-33141692	-	LINC00486; LOC100271832 (upstream 10.501 kb); TTC27 (downstream 95.162 kb)
20863	52	6	1884-2429	E1	-	chr2: 33141285-33141624	-	LINC00486; LOC100271832 (upstream 10.569 kb); TTC27 (downstream 95.167 kb)
20863	48	9	7182-7781	URR	-	chr2: 33141295-33141692	-	LINC00486; LOC100271832 (upstream 10.501 kb); TTC27 (downstream 95.177 kb)
20863	39	6	4865-5317	L2	-	chr2: 33141298-33141679	-	LINC00486; LOC100271832 (upstream 10.514 kb); TTC27 (downstream 95.18 kb)
20863	41	8	36-583	URR/E6/E7	-	chr2: 33141299-33141662	-	LINC00486; LOC100271832 (upstream 10.531 kb); TTC27 (downstream 95.181 kb)
20863	44	11	4231-4810	L2	-	chr2: 33141302-33141692	-	LINC00486; LOC100271832 (upstream 10.501 kb); TTC27 (downstream 95.184 kb)
20863	27	7	776-1370	E7/E1	+	chr2: 33141312-33141692	-	LINC00486; LOC100271832 (upstream 10.501 kb); TTC27 (downstream 95.194 kb)
20863	45	4	3398-3629	E2/E4	-	chr2: 33141327-33141680	-	LINC00486; LOC100271832 (upstream 10.513 kb); TTC27 (downstream 95.209 kb)
20863	40	7	6010-6406	L1	-	chr2: 33141341-33141679	-	LINC00486; LOC100271832 (upstream 10.514 kb); TTC27 (downstream 95.223 kb)
20863	29	7	4253-4822	L2	+	chr2: 33141417-33141670	-	LINC00486; LOC100271832 (upstream 10.523 kb); TTC27 (downstream 95.299 kb)
20863	30	5	2410-3029	E1/E2	+	chr2: 33141419-33141678	-	LINC00486; LOC100271832 (upstream 10.515 kb); TTC27 (downstream 95.301 kb)
20863	37	14	5390-5960	L2/L1	-	chr2: 33141421-33141692	-	LINC00486; LOC100271832 (upstream 10.501 kb); TTC27 (downstream 95.303 kb)
20863	23	4	7471-7894	URR	+	chr2: 33141425-33141663	-	LINC00486; LOC100271832 (upstream 10.53 kb); TTC27 (downstream 95.307 kb)
20863	32	5	230-815	E6/E7	+	chr2: 33141430-33141670	-	LINC00486; LOC100271832 (upstream 10.523 kb); TTC27 (downstream 95.312 kb)
20863	31	5	3048-3796	E2/E4	+	chr2: 33141446-33141672	-	LINC00486; LOC100271832 (upstream 10.521 kb); TTC27 (downstream 95.328 kb)
20863	42	10	2798-3514	E1/E2/E4	-	chr2: 33141449-33141692	-	LINC00486; LOC100271832 (upstream 10.501 kb); TTC27 (downstream 95.331 kb)
20863	43	5	1299-1596	E1	-	chr2: 33141457-33141688	-	LINC00486; LOC100271832 (upstream 10.505 kb); TTC27 (downstream 95.339 kb)
20863	51	6	6531-6940	L1	-	chr2: 33141468-33141648	-	LINC00486; LOC100271832 (upstream 10.545 kb); TTC27 (downstream 95.35 kb)
20863	24	4	5752-6286	L1	+	chr2: 33141477-33141653	-	LINC00486; LOC100271832 (upstream 10.54 kb); TTC27 (downstream 95.359 kb)

20863	46	4	2511-2864	E1/E2	-	chr2: 33141524-33141690	-	LINC00486; LOC100271832 (upstream 10.503 kb); TTC27 (downstream 95.406 kb)
20863	25	4	6354-6693	L1	+	chr2: 33141527-33141692	-	LINC00486; LOC100271832 (upstream 10.501 kb); TTC27 (downstream 95.409 kb)
20863	59	8	2788-3018	E1/E2	+	chr3: 186507881-186508213	-	RFC4; EIF4A2 (downstream 0.196 kb); SNORA4 (downstream 2.343 kb); SNORA63 (downstream 2.659 kb); SNORA81 (downstream 3.240 kb); MIR1248 (downstream 3.315 kb); SNORD2 (downstream 5.227 kb); ADIPOQ (upstream 52.249 kb)
20863	66	5	4174-4363	L2	-	chr8: 38059390-38059439	+	BAG4; LSM1 (upstream 25.142 kb); PPAPDC1B (downstream 61.21 kb)
20863	10	5	4178-4491	L2	-	chr11: 57805154-57805208	+	OR9Q1; OR6Q1 (downstream 5.776 kb); OR9Q2 (upstream 152.697 kb)
20863	13	7	4187-4722	L2	-	chr12: 66451369-66451445	+	MIR6074 (upstream 33.863 kb); LLPH (downstream 65.403 kb)
20863	14	11	4177-4618	L2	-	chr12: 80007753-80007807	+	PAWR; MIR5692B (downstream 155.313 kb)
20863	15	5	4174-4444	L2	-	chr15: 32767594-32767649	-	GOLGA8O (upstream 19.759 kb); WHAMMP1 (downstream 44.399 kb)
20863	17	7	6645-6877	L1	-	chr17: 47285978-47286154	+	GNGT2; ABI3 (upstream 1.434 kb); PHOSPHO1 (downstream 14.577 kb)
20863	20	4	4179-4470	L2	-	chr19: 7124552-7124609	-	INSR; ZNF557 (downstream 36.574 kb); MBD3L3 (upstream 65.907 kb)
20863	56	4	6420-6639	L1	-	chr20: 15285794-15286003	-	MACROD2; MACROD2-AS1 (upstream 375.63 kb)
20863	57	12	4176-4459	L2	-	chr20: 52118023-52118075	-	TSHZ2 (downstream 6.154 kb); LOC101927770 (upstream 51.233 kb)
20863	75	5	4202-4689	L2	-	chrX: 113975931-113975990	-	HTR2C; MIR1911 (upstream 21.753 kb); MIR1298 (downstream 26.17 kb)
