# Supplementary material for: HPV integration hijacks and multimerizes a cellular enhancer to generate a viral-cellular super-enhancer that drives high viral oncogene expression
Source: PLoS Genet. 2018 Jan 24;14(1):e1007179. doi: 10.1371/journal.pgen.1007179 (PMC5798845; doi:10.1371/journal.pgen.1007179)
Supplement: S2 Table — (PDF) [file pgen.1007179.s009.pdf]

S2 Table. Primer sequences used for APOT assay, ChIP-qPCR and generation of FISH probe

Primer	Sequence (5'->3')	Purpose	Product length (bp)
Amplicon 1F	TAAACGAGGGCAGAGAGAGC	Probe against 20861 amplified cellular flanking sequence	2,184
Amplicon 1R	CAGGCCCTGTTCTAAGCACT		
Amplicon 2F	CCCAGAATCTGAGCCAAGCA	Probe against 20861 amplified cellular flanking sequence	2,191
Amplicon 2R	TCATCCCCGGGAATCTGACT		
Amplicon 3F	AAAACAGGCTGGTGGGAACA	Probe against 20861 amplified cellular flanking sequence	2,473
Amplicon 3R	TTTTGCCCCCACTGACAACT		
Amplicon 4F	TGTTTCCCTATCCACGGTGC	Probe against 20861 amplified cellular flanking sequence	2,442
Amplicon 4R	GTGGGGTCATGACTTGGAGG		
Amplicon 5F	CCGATGCTGTTGATTAGAGGC	Probe against 20861 amplified cellular flanking sequence	2,377
Amplicon 5R	ATTGGGCACCACACATTTGC		
Amplicon 6F	ACCTGTCTCTCTCCCCATCC	Probe against 20861 amplified cellular flanking sequence	2,396
Amplicon 6R	CCTCTCTGCCCCAAACAGAG		
Amplicon 7F	GATCATTGAACGAGGCACGC	Probe against 20861 amplified cellular flanking sequence	2,497
Amplicon 7R	TGCCTTGTAATCAGGGGCAG		
Amplicon 8F	CTGCGAGCTCTGAGAGAGAC	Probe against 20861 amplified cellular flanking sequence	2,035
Amplicon 8R	TGGTGGCTCTGTTATCTGCG		
Amplicon 9F	CTGTGTGTCTTTAGGGGCGT	Probe against 20861 amplified cellular flanking sequence	2,181
Amplicon 9R	TTGATGGGATTGCCTGGGAC		
APOT 20861_P1	CGGACAGAGCCCATTACAAT	To determine viral-cellular junction transcript in 20861 cells by amplification of papillomavirus oncogene transcripts, APOT (see (30))	-
Linker sequence (P3)	GACTCGAGTCGACATCG		
APOT 20861_P2	CCTTTTGTTGCAAGTGTGACTCTACG		
Oligo(dT)17-P3	GACTCGAGTCGACATCGATTTTTTTTTTTTTTTTT		
Flank seq F	TCGCCCACCAGCCTTAGCA	To determine copy number of cellular flanking sequence at chr2 20861 integration site	114
Flank seq R	AATGCCAGCTGATAAATGTAGAGTGAGTG		
ACTB F	TCGTCCACCGCAAATGC	Normalization gene; copy number analysis of cellular flanking sequence at chr2 20861 integration site	76
ACTB R	CGCAAGTTAGGTTTTGTCAAGAAA		
URR F	TTCGGTTGCATGCTTTTTGGCACAA	ChIP-qPCR	97
URR R	CACGCATGGCAAGCAGGAAACGTAC		
L1 F	GGCTGCCTAGTGAGGCCACTG	ChIP-qPCR	79
L1 R	GCGTGCAACATATTCATCCGTGC		
E2 F	CGCCGCGACCCATACCAAAG	ChIP-qPCR	93
E2 R	GGGGTTTCCGGTGTCTGGCT		
IGLL5 F	CTTGAGGATTGCAGATGGGC	ChIP-qPCR; negative control for super-enhancer	77
IGLL5 R	TGACCCTGATCCTGACCCTA		
CCND2 F	CCTTCTGCTCCACCTTCTCT	ChIP-qPCR; positive control for super-enhancer	76
CCND2 R	CTGACCTCCTTCCTTTGGCT		
FOSL1 F	AACGTCCCTGTTCCCATTCT	ChIP-qPCR; positive control for super-enhancer	92
FOSL1 R	GGCAGAGCTAGAACCCACTT		
PPIA F	AGAACTTCATCCTAAAGCATACGG	RT-qPCR; normalization gene	121
PPIA R	TGCTTGCCATCCAACCACTC		
E6*I F	ACAGTTACTGCGACGTGAGGTG	RT-qPCR	56
E6*I R	TTCTTCAGGACACAGTGG		
20861 fusion F	TCTACCATGGCTGATCCTGCAGGTATGT	RT-qPCR; viral-host fusion mRNA at chr2 integration site in 20861 cells	105
20861 fusion R	GAGTCATCAAACGATGCTAAGGCTGGTG		
20831/62 fusion F	GCTTTGTAATCAGATGGGATATTATG	RT-qPCR; viral-host fusion mRNA at chr3 integration site in 20831 and 20862 cells	84
20831/62 fusion R	TTGTACATTGTATACTGCAGTGTCGT		
